# Differential Interactome Proposes Subtype-Specific Biomarkers and Potential Therapeutics in Renal Cell Carcinomas

**DOI:** 10.3390/jpm11020158

**Published:** 2021-02-23

**Authors:** Aysegul Caliskan, Gizem Gulfidan, Raghu Sinha, Kazim Yalcin Arga

**Affiliations:** 1 Department of Bioengineering, Marmara University, Istanbul 34722, Turkey; gulaysecaliskan@hotmail.com (A.C.); gizemgulfidn@gmail.com (G.G.); 2 Faculty of Pharmacy, Istinye University, Istanbul 34010, Turkey; 3 Department of Biochemistry and Molecular Biology, Penn State College of Medicine, Hershey, PA 17033, USA

**Keywords:** renal cancers, protein interactome, diagnostic biomarker, prognostic biomarker, virtual screening, docking

## Abstract

Although many studies have been conducted on single gene therapies in cancer patients, the reality is that tumor arises from different coordinating protein groups. Unveiling perturbations in protein interactome related to the tumor formation may contribute to the development of effective diagnosis, treatment strategies, and prognosis. In this study, considering the clinical and transcriptome data of three Renal Cell Carcinoma (RCC) subtypes (ccRCC, pRCC, and chRCC) retrieved from The Cancer Genome Atlas (TCGA) and the human protein interactome, the differential protein–protein interactions were identified in each RCC subtype. The approach enabled the identification of differentially interacting proteins (DIPs) indicating prominent changes in their interaction patterns during tumor formation. Further, diagnostic and prognostic performances were generated by taking into account DIP clusters which are specific to the relevant subtypes. Furthermore, considering the mesenchymal epithelial transition (MET) receptor tyrosine kinase (PDB ID: 3DKF) as a potential drug target specific to pRCC, twenty-one lead compounds were identified through virtual screening of ZINC molecules. In this study, we presented remarkable findings in terms of early diagnosis, prognosis, and effective treatment strategies, that deserve further experimental and clinical efforts.

## 1. Introduction

Kidney cancer is among the 10 most common cancers in adults and renal cell carcinoma (RCC) shows a steady increase in prevalence [1]. RCC is known to be the most common type of kidney cancer and is responsible for up to 85% of cases; it is more common in males than in females (ratio, 1.7:1), and most of the patients are at an older age (average age of 64 years) [1]. Primarily, RCC is categorized into subtypes according to histological classification under a microscope, including clear cell (ccRCC, also known as KIRC), papillary (pRCC, also known as KIRP), chromophobe (chRCC, also known as KICH), and some other, less common subtypes such as collecting duct, medullary RCC, and unclassified RCC [2]. The most prevalent one among kidney cancers is ccRCC which represents 75–80% of RCC [3] and derives its name from its clear cytoplasm on the pathologic analysis [4]. The rest are papillary (10–15%), chromophobe (5%), and rare kidney cancers. Although improvement of the state-of-the-art treatment technologies, the overall prognosis is still poor in RCCs, particularly for patients who present with the advanced-stage disease [1]. Therefore, early diagnosis and successful urological procedures with partial or total nephrectomy can be life-saving. However, only about 10% of RCC patients present with urological problems or other known clinical symptoms. More than sixty percent of patients are incidentally noticed at imaging investigations [5], and metastasis has already begun in nearly 20–30% of the patients when diagnosed [6]. In this context, biomarker identification from secretion fluids is extremely important for early diagnosis. Furthermore, biomarkers are becoming increasingly significant to facilitate the discovery of anti-cancer agents, to distinguish cancer cells from the other cells, to understand drug action mechanisms, to predict prognosis, to design personalized medication, and to understand the mechanisms underlying response to therapy. All types of kidney cancers are different in many respects including tumor location within the kidney, the cell type from which they originate, and alterations on their genotype, making it even more crucial to characterize the pathology of each type and to identify specific proteins as druggable targets.

Biomarkers play an important role in the implementation of personalized medicine in clinics with respect to defining subtype phenotypes, predicting clinical course and prognosis, and determining the appropriate therapeutic approach. In this respect, a comprehensive pool of molecular markers from different biological levels (hub proteins, receptors, miRNAs, mRNAs, reporter TFs, and metabolites) were presented from a systematic integrative biology perspective with the potential to provide in-depth knowledge into the disease mechanisms in RCC subtypes [7]. On the other hand, the limited diagnostic and prognostic performance of a molecular biomarker revealed the need for system biomarkers to be obtained with approaches that consider interactions between critical molecules such as the differential protein interactome [8,9].

The differential interactome methodology is based on the idea that significant alterations occur in the protein–protein interactions (PPIs) among phenotypes. The success of this approach has been effectively demonstrated in various cancers and their subtypes [8,9,10]. The differential interactome approach made it possible to estimate the probability distributions for any possible co-expression profile of gene pairs (encoding proteins that interact with each other) across phenotypes and to determine the uncertainty of whether a PPI is meeting the corresponding phenotype.

The Cancer Genome Atlas (TCGA) is one of the comprehensive cancer genomics datasets available. The availability of TCGA allows researchers to uncover the molecular profiling of tumors through the application of genome analysis technologies, including large-scale genome sequencing. In our present study, we investigated the TCGA transcriptome data from 892 individuals and used the differential interactome methodology [8] that integrates transcriptome data with the human protein interactome network to analyze and compare the differential protein–protein interactions among healthy and tumor groups. Three common subtypes (ccRCC, pRCC, and chRCC) of RCC were investigated and compared in terms of the differential interactome profiles. These analyses allowed us to identify differentially interacting proteins (DIPs) that represent significant changes in their interaction patterns during the transition from “normal” to “tumor” phenotypes and are therefore differently related to the corresponding tumor [9]. We also determined candidate protein panels with high diagnostic and/or prognostic performance, which might allow us to develop novel drug candidates and to diagnose patients in the early stage. Furthermore, we offer drug candidates that showed an inhibitory effect on mesenchymal epithelial transition (MET) receptor tyrosine kinase which is one of the DIPs that have activated interactions in the case of pRCC.

## 2. Materials and Methods

### 2.1. Collecting of Gene Expression Data

The transcriptome datasets consisting of three different subtypes of kidney cancer (chRCC, ccRCC, and pRCC) were acquired from the TCGA database [11] to analyze their gene expression profiles. The number of the primary tumor and the matched normal tissue samples were 538 and 72 for ccRCC, 289 and 32 for pRCC, and 65 and 24 for chRCC, respectively.

### 2.2. Obtaining Protein–Protein Interactions Data

Physical PPI data experimentally detected in humans was obtained from the BioGRID database using the latest version (v. 4.0.189) [12]. The data contained 51,745 PPIs among 10,177 human proteins. After filtering the PPI data for proteins encoded by genes having transcriptome data in TCGA datasets, a network was reconstructed with 34,604 PPIs among 8322 proteins.

### 2.3. Identification of Differential Interactome and Differentially Interacting Proteins

The gene expression profiles of RCC subtypes were analyzed together with the obtained PPI data through the differential interactome algorithm revised in the study of Gulfidan et al. [8] using R (version 3.6.1). This algorithm presents the differential PPIs (dPPIs) between the tumor phenotype and normal phenotype, taking into account the relative observation frequencies (*q*-value) of each PPI as described earlier [8,9]. The criteria of the algorithm for obtaining significant dPPIs were set as *q*-value < 0.10 (significantly repressed in tumor phenotype), *q*-value > 0.90 (significantly activated in tumor phenotype), and a normalized observation frequency either in normal or tumor phenotype > 20%.

DIPs, the proteins having differential interactions, were classified into two groups according to their interaction patterns: (i) DIPs having repressed interactions under tumor condition, and (ii) DIPs having activated interactions under tumor condition. DIPs that were specific to the RCC subtypes and were common in all subtypes were detected for further analyses. The networks consisting of dPPIs and DIPs were visualized through the Cytoscape 3.4.0 [13].

### 2.4. Evaluation of the Secretion Levels of Subtype-Specific DIPs in Body Fluids

The secretion levels (ppm) of subtype-specific DIPs in plasma, serum, urine, and saliva were investigated through protein expression data which is accessible in the GeneCards [14] database curating the proteomics databases; ProteomicsDB [15], MaxQB [16], and MOPED [17].

### 2.5. Analysis of Diagnostic Performance and Prognostic Power

Principal component analyses (PCA) were carried out for the assessment of the diagnostic potential of subtype-specific DIPs using the expression values of genes encoding the DIPs which had the secretion levels in body fluids. The simulations were performed using the gene expression data of tumor samples of ccRCC, pRCC, and chRCC datasets for each subtype-specific DIPs, separately.

To explore the prognostic performance of each subtype-specific DIP, survival analyses were carried out through stratification of patients into high- and low-risk groups based on their prognostic index (PI), which is the linear component of the Cox model (PI = β_1_x_1_ + β_2_x_2_ +…+ β_p_x_p_, where β_i_ is coefficient acquired from the Cox fitting, x_i_ is the expression value of each gene). Analyses were implemented through the SurvExpress tool [18] utilizing two RNA-Seq originated datasets of ccRCC with 415 samples, and pRCC with 278 samples including clinical data. In addition, RNA–Seq originated chRCC dataset with 9 samples with clinical data retrieved from TCGA [11] was analyzed separately through the pipeline established in our previous study [8] due to the absence of any dataset related to the chRCC subtype in the SurvExpress database. The signatures of survival in each risk group were estimated by Kaplan–Meier curves and Hazard Ratios (HR). Statistical significance of each plot was evaluated by the cut-off for log-rank *p*-value < 0.05. Hazard ratio (HR = O_1_/E_1_/O_2_/E_2_) was calculated to discover the significance of the survival curves based on the ratio between the relative death rate in group 1 (O_1_/E_1_) and the relative death rate in group 2 (O_2_/E_2_), where O denotes the observed number of deaths, and E denotes the expected number of deaths.

### 2.6. Identification of Candidate Drugs through Virtual Screening

We set the following criteria to determine the potential drug target protein among DIPs in docking studies: (i) its interactions should be activated in the disease state, and (ii) it should have at least 5 interactions. Among DIP proteins of pRCC, MET protein satisfied all the criteria and came to the forefront as a potential drug target. Through virtual screening, potential molecules targeting MET were determined. To have an insight into the ligand-receptor interactions, the available X-ray crystal structures of MET were fetched from the Protein Data Bank (PDB) (www.rcsb.org) [19]. PDB entry 3DKF was chosen for all the docking studies according to the resolution, Ramchandran outliers, and structural similarity between the screened ligands and the co-crystallized ligands. Virtual Screening binding analysis was carried out on the assigned binding site of the X-ray crystal structure of MET [20] exploiting ZINC molecules described by the publicly available ZINC15 library [21]. Molecular docking studies were executed for 703 substances retrieved from the ZINC15 library through AutoDock Vina [22] in the PyRx virtual screening tool (v. 0.8) [23].

## 3. Results

### 3.1. Differential Interactome Estimation in Subtypes of RCC 

**Figure 1:**
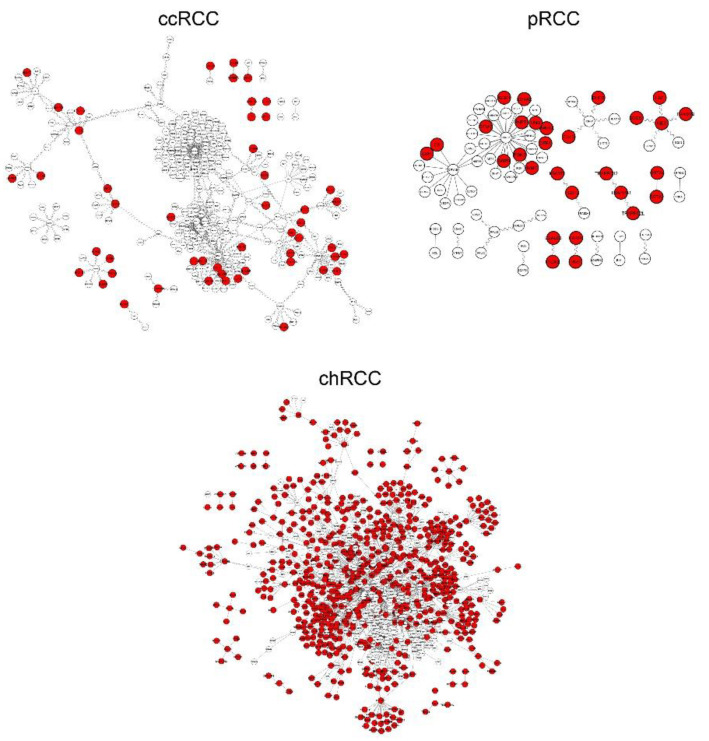
Differential interactome networks reconstructed with differential protein–protein interactions (dPPIs) around differentially interacting proteins (DIPs) in three Renal Cell Carcinoma RCC subtypes. Red nodes represent DIPs specific to the subtype of interest. ccRCC: Clear Cell Renal Carcinoma; pRCC: Papillary Renal Cell Carcinoma; chRCC: Chromophobe Renal Cell Carcinoma.

RNA-seq transcriptome data of three RCC subtypes were retrieved from TCGA to apply differential interactome methodology [8] for prediction of highly probable PPIs in each state and identification of differential PPIs. To this end, we examined transcriptomic data for three common subtypes of RCC with an adequate number of samples (*n* > 24) in both normal and tumor groups (see Materials and Methods section). The scale-free topology of the differential interactome network brings out the presence of hubs called DIPs indicating substantial changes in their interaction patterns during the transition from “normal” to “tumor” phenotypes [8,9]. We determined 628 DIPs for chRCC, 50 DIPs for ccRCC, and 29 DIPs for pRCC as subtype-specific DIPs, whereas 33 DIPs were common in all subtypes (Supplementary Table S1). The tumor-specificity of DIPs varied according to the subtype (Figure 1).

Further analyses (i.e., determination of prognostic power, diagnostic performance, and druggability) were implemented by taking into account 50 DIPs specific to ccRCC, 29 DIPs specific to pRCC, and the top 50 DIPs having the most interactions specific to chRCC (Table 1). We considered those DIPs as a cluster for each subtype and suggested them as potential systems biomarkers for the development of effective diagnosis, prognosis, and treatment strategies.

**Figure 2:**
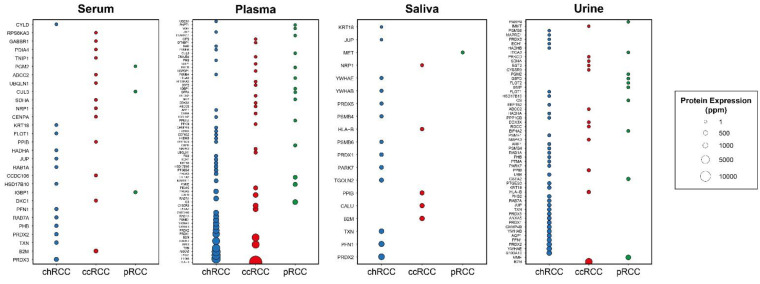
Bubble plots indicating protein expression levels of DIPs specific to three subtypes in different body fluids including serum, plasma, saliva, and urine. The x-axis indicates subtypes while the y-axis indicates protein symbols.

Then, we filtered DIPs by considering whether they are secreted in body fluids and renamed secreted proteins as “s-DIPs” (Table 1, Figure 2). s-DIPs represent proteins that were expressed at least in one of the following media: serum, plasma, saliva, or urine (www.genecards.org) [14]. The importance of secretion in body fluids that can be accessed without surgery is that it might provide serious convenience for early diagnosis. While s-DIPs were used for diagnosis analysis, all DIPs (s-DIPs and non-s-DIPs) were considered in prognosis and druggability (virtual screening) analyses.

### 3.2. Prognostic and Diagnostic Capabilities of DIPs Clusters

We considered the clusters of DIPs as potential systems biomarkers for each RCC subtype and analyzed their diagnostic performance and prognostic power. 

**Figure 3:**
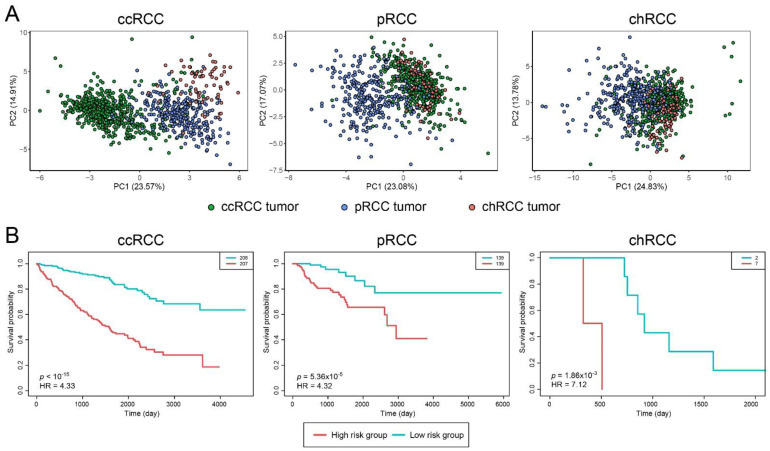
Diagnostic and prognostic performance analysis results for Renal Cell Carcinoma (RCC) subtypes. (**A**) Principal component analyses (PCA) plots, visualized by considering s-DIPs, indicating the individual differences in the gene expression profiles in tumor samples among the subtypes. (**B**) Kaplan–Meier curves estimating patients’ survival for three subtypes based on categorization of patients into high- and low-risk groups via prognostic index. ccRCC: Clear Cell Renal Carcinoma; pRCC: Papillary Renal Cell Carcinoma; chRCC: Chromophobe Renal Cell Carcinoma; HR: Hazard Ratio; PC: Principal component.

The diagnostic analysis was performed via PCA using s–DIPs (Table 1). All s–DIP clusters exhibited significantly high diagnostic performance for relevant subtypes (Figure 3A).

Prognostic capabilities of gene clusters were quantified through log-rank p-values and visualized by Kaplan–Meier curves (Figure 3B). Cox (proportional hazards) regression was also engaged to estimate HRs. These analyses were carried out utilizing TCGA clinical datasets (see Materials and Methods section). Gene clusters were significantly predictive in terms of patient survival risk assessment for the respective subtype (Figure 3B, ccRCC *p* < 1 × 10^−15^, pRCC *p* = 5.36 × 10^−5^, chRCC *p* = 1.86 × 10^−3^). Through Cox-proportional hazard analysis, HR values were estimated as 4.33, 4.32, and 7.12 for ccRCC, pRCC, and chRCC, respectively.

### 3.3. Discovery of Drug Candidates through Virtual Screening Analyses

In silico simulation techniques have become an indispensable tool for modern-day drug discovery programs. Molecular docking currently offers the best alternative to quickly estimate the binding conformations of ligands that are energy-efficient to interact with a pharmacological receptor site. It has become more popular as it is time and cost effective in the pipeline of drug discovery and development. Interactions of some DIP proteins within the module were activated during the tumorigenesis, while some were found to be repressed. We hypothesized that, if we manage to break through the interactions that are activated, we might model a strategy to cure the disease. For this purpose, we considered DIPs with activated interactions in the tumor state as potential drug targets.

**Figure 4:**
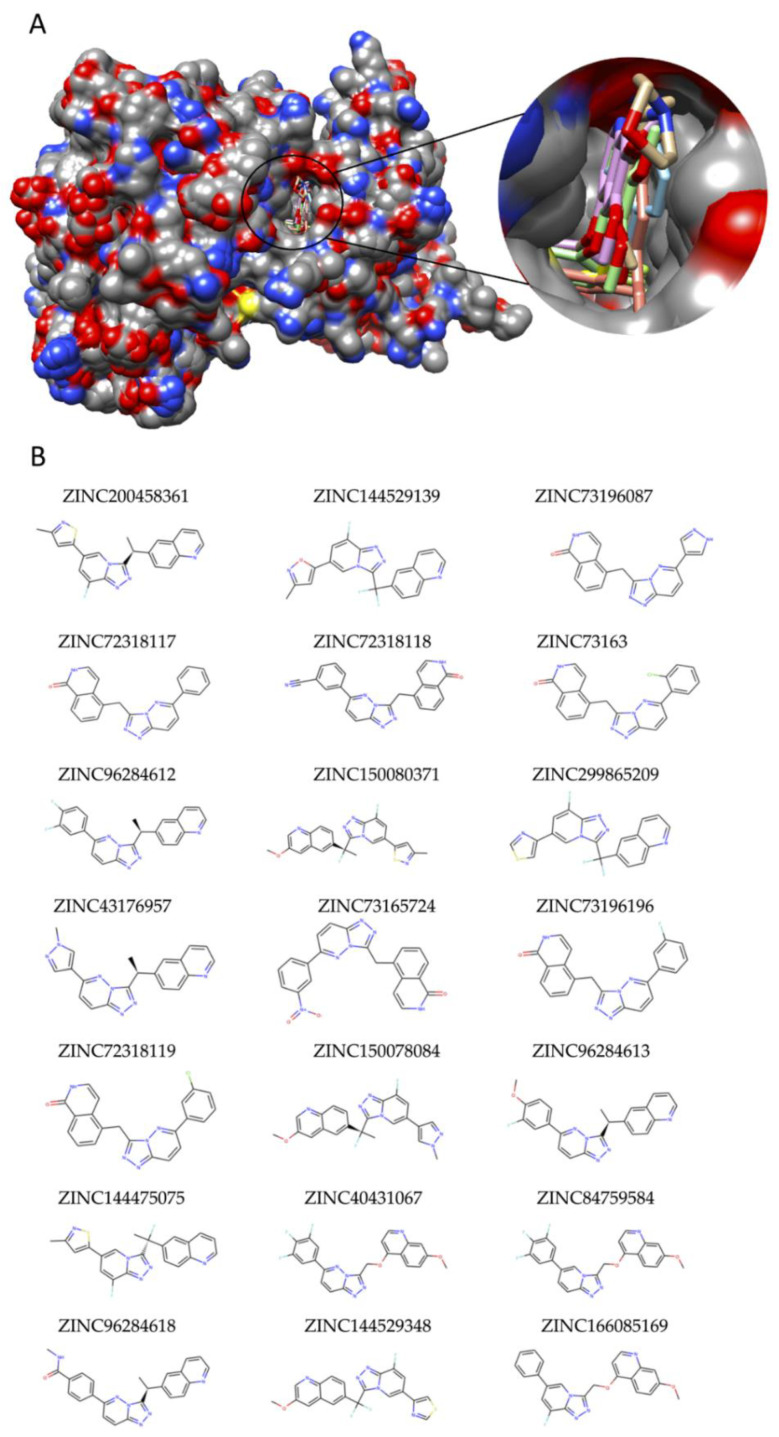
Virtual screening to identify potential hit drug candidates for pRCC. (**A**) Superposition of X-ray crystal structures of MET retrieved from RCSB for the validation of docking protocol. (**B**) 2D structures of ZINC molecules that showed high binding affinities to MET protein in virtual screening.

For instance, among DIP proteins of the pRCC subtype, MET protein came into prominence as a potential drug target. Candidate molecules targeting MET were determined via virtual screening of the ZINC15 library via the available crystal structures of MET from PDB. All available X-Ray crystal structures of MET (PDB IDs: 3DKF, 2RFN, 3EFJ, 3U6H, 4EEV) and their bound ligands were superposed, and potential binding sites were determined to identify the binding site location on the receptor (Figure 4A). Virtual Screening binding analysis was accomplished on the assigned binding site of the X-ray structure of MET (PDB ID: 3DKF) utilizing ZINC molecules which were described by the ZINC15 library. The virtual screening analysis revealed twenty-one ZINC molecules with high binding affinities (ΔG^0^ ≤ −12, LE > 0.35) (Table 2; Figure 4B).

## 4. Discussion

Dysregulations in various biochemical pathways play an important role in cancer formation and development. Genetic studies have identified numerous molecular defects in cancer cells and suggested multiple potential targets for therapeutic intervention. Conventional drug design has mainly focused on the inhibition of a single protein, usually an enzyme or receptor; however, this strategy has not been successful enough, as the development and progression of cancers are mostly due to the coordinated action of a group of biological entities rather than a single molecule dysfunction [24]. Hereby, PPIs have become highly promising targets that cover many therapeutic areas and potential intervention points for the development of anticancer agents. Until now, significant progress has been made in identifying small molecule inhibitors of various protein–protein systems in the field of oncology, and powerful and selective drug-like molecules that inhibit many interactions such as p53-MDM2 interaction have been discovered [25]. Furthermore, a number of these small-molecule inhibitors, such as Siremadlin, AMG-232, and APG-115 have progressed to early phase clinical trials [26].

Our study reports the generation of the dPPI networks in RCC subtypes through the implementation of high throughput transcriptome and protein interactome data. The integration of respective RNA-seq datasets and differential interactome approach allowed the identification of dPPIs in different conditions (tumor/normal) in RCC subtypes. The study unveils and compares the dPPIs for each subtype and identifies DIPs through a differential interactome. Further analyses on DIPs may be useful in understanding the tumor mechanisms. For instance, our findings revealed that HspB1 protein is one of the common DIPs for three subtypes. The correlation between HspB1 expression in RCC subtypes and metastasis process has been revealed in previous studies and HspB1 is known to facilitate metastasis by suppressing anti-cancer response such as apoptosis and senescence [7,27].

DIP clusters were used for diagnostic and prognostic analyses for each subtype. Despite the improvements in the state of the art treatment technologies, the overall prognosis is still poor in RCCs and more than 50% of RCCs are diagnosed incidentally [28]. Even the detection of the early asymptomatic stage during routine examination could have a profound impact on clinical outcome. Therefore, an effective, clinically useful test for early detection of RCC subtypes should be measurable in readily accessible body fluids, such as plasma, serum, urine, or saliva. For this purpose, we filtered DIPs by considering whether they are expressed in those body fluids at the protein level and defined the s-DIP concept here for the first time in literature. s-DIP clusters characterize patients well in terms of the diagnostic group (subtype) to which they belong. Hence, we offer that s-DIPs might be used for the diagnosis of candidate RCC patients after further experimental and clinical validations. 

Saliva is one of the complex and important multi-constituent body fluids that reflects a wide variety of physiological knowledge due to its contents extensively supplied by the blood. Moreover, a saliva-based diagnosis has been drawing attention in the diagnosis of systemic diseases such as renal cancers, due to the source, composition, function, and interaction of saliva with the substances that make up the plasma [29,30]. In the present study, besides blood components and urine, we also demonstrated the potential of saliva as a non-invasive potential media for RCC diagnosis, especially in chRCC.

The three basic elements for the art of medicine are diagnosis, therapeutics, and prognosis. Therefore, after making the correct and early diagnosis, determining the optimal treatment strategies would be important and as a follow-up, one could provide up-to-date information on the patient’s prognosis. Our present investigation also aimed to provide new targets for the design of novel therapies in RCC subtypes and putative biomarkers with prognostic significance. In this study, DIP clusters appear to be strong putative candidates for the prognostic marker in each related subtype. Survival analyses through stratification of patients according to clinicopathological variables such as tumor stage or grade would demonstrate the prognostic power of the potential biomarkers better. However, despite the presence of comprehensive gene expression profiling efforts such as TCGA, transcriptome data with available clinical information is still limited for RCCs, even for the most common subtypes.

Additionally, to shed light on the further experimental studies, we identified MET protein as an ideal potential drug target in pRCC and showed the high potential of twenty-one Zinc molecules (Table 2) as candidate therapeutics for future preclinical studies. The integration of the transcriptome and protein interactome data with the drug knowledge helped to uncover 21 in silico validated potential drug candidates for pRCC. These in-silico findings can be used further to design and synthesize novel MET inhibitors. Furthermore, ZINC73196087, ZINC72318117, ZINC72318118, ZINC73163075, ZINC73165724, ZINC73196196, and ZINC72318119 have been shown to demonstrate effective anti-proliferative activity against a panel of c-Met-amplified gastric cancer cell lines [31]. We propose that these ZINC compounds should also be evaluated with experimental studies for RCC cell lines and we conclude that these molecules might be potential therapeutics for the management of the pRCC. Further in vitro/in vivo pharmacological evaluations and clinical validations are needed for approval of these candidate drugs.

The major limitation of the study is the lack of experimental validations of the identified ZINC compounds on the RCC samples or cell lines. Future in vitro studies need to be conducted to evaluate the effects of ZINC compounds identified on cell viability, proliferation, and migration. Moreover, the mechanism of actions of these molecules need to be investigated in detail to elucidate their effect on molecular pathways such as apoptosis and cell cycle. Rather than being considered as a single agent, these compounds can also be regarded as adjuvant therapy to the baseline therapeutics, then, the critical extension of this work would be to learn whether the observations of in vitro studies can be recapitulated by in vivo studies and eventually in clinical trials. Another point that has a crucial role in translation to the clinic is sampling where body fluids are favorable for the detection of the biomarkers. Proteomics studies also need be verified for the proteins exhibiting significantly high diagnostic and prognostic performance for relevant subtypes. Moreover, these biomarkers could also assist oncologists to assist in optimal diagnosis and prognosis management.

## Figures and Tables

**Table 1:** Differentially interacting proteins (DIPs) specific to RCC subtypes.

Specificity	s-DIPs ^1^	Non s-DIPs ^2^
ccRCC-specific	ABCC2, B2M, BST2, CALU, CCDC106, CENPA, CYB5R3, DDX3X, DKC1, DNAJB4, DTNBP1, GABBR1, GIT2, HLA-B, HSPBP1, IMMT, MAPK3, NRP1, PDIA4, PEA15, PFDN2, PFKM, PPIB, PRKCD, RGCC, RPS6KA3, SDHA, UBQLN1, TNIP1	AZIN1, CDT1, ELF4, FBXW8, GPS2, IL32, IRF1, LDOC1, MCM7, MCM9, MTF1, MTOR, P4HA2, PHLPP1, RSL1D1, SCD, TAF1, TAPBP, TOMM20, USP2, ZNF668
pRCC-specific	CS, CUL3, DFFA, DHFR, EIF4A2, FLOT2, G6PD, GSTA2, IGBP1, ITGA3, MET, MME, MVP, PARP4, PGM2, PNPT1, PPM1A, TRAPPC1	GSTA4, HGF, LBH, LGALS8, MMGT1, RANBP9, SF3A3, SOCS1, TRAPPC12, TRAPPC2L, UNG
chRCC-specific	ANXA5, AQP1, ARF1, BAD, CHMP4B, CYLD, ECH1, EEF1B2, FLOT1, FUS, HADHA, HADHB, HSD17B10, JUP, KRT18, MAPRE1, PARK7, PFN1, PHB, PHB2, PPP1CB, PRDX1, PRDX2, PRDX3, PRDX5, PSMB4, PSMB6, PSME1, PTGES3, PTMA, RAB1A, RAB7A, S100A10, TGOLN2, TXN, UBB, UBE3A, YWHAB, YWHAE	ABL1, AMFR, ARAF, CDK9, FOS, JUND, MCL1, MORF4L2, SF3B5, STAU1, TRIM8

^1^ Protein expression was observed at least in one of the following body fluids: serum, plasma, saliva, urine; ^2^ Protein expression was not observed in any of the following body fluids: serum, plasma, saliva, urine.

**Table 2:** The ZINC molecules presented the best binding affinities to MET.

Ligand ZINC15 ID	Vina Binding Affinity (kcal/mol)	Ligand Efficiency (LE)
ZINC200458361	−12.7	0.41
ZINC144529139	−12.6	0.39
ZINC73196087	−12.6	0.45
ZINC72318117	−12.5	0.44
ZINC72318118	−12.5	0.41
ZINC73163075	−12.5	0.42
ZINC96284612	−12.5	0.41
ZINC150080371	−12.4	0.38
ZINC299865209	−12.4	0.42
ZINC43176957	−12.4	0.43
ZINC73165724	−12.4	0.39
ZINC73196196	−12.4	0.43
ZINC72318119	−12.3	0.41
ZINC150078084	−12.2	0.37
ZINC96284613	−12.2	0.39
ZINC144475075	−12.1	0.4
ZINC40431067	−12.1	0.37
ZINC84759584	−12.1	0.36
ZINC96284618	−12.1	0.37
ZINC144529348	−12	0.41
ZINC166085169	−12	0.38

## Data Availability

Publicly available datasets were analyzed in this study. Transcirptome data can be found here: [https://portal.gdc.cancer.gov/, Primary site: Kidney]. Protein interactome data is available here: [https://thebiogrid.org, File repository: BIOGRID-4.0.189].

## Supplementary Materials

The following are available online at https://www.mdpi.com/2075-4426/11/2/158/s1, Table S1: List of Differentially Interacting Proteins (DIPs) in chRCC, ccRCC, pRCC.

## Abbreviations

ccRCC	Clear Cell Renal Cell Carcinoma
chRCC	Chromophobe Renal Cell Carcinoma
DIP	Differentially interacting protein
dPPI	Differential protein–protein interaction
HR	Hazard ratio
KICH	Kidney Chromophobe
KIRC	Kidney Renal Clear Cell Carcinoma
KIRP	Kidney Renal Papillary Cell Carcinoma
LE	Ligand efficiency
ns-DIP	Non-secreted DIP
PC	Principle component
PCA	Principal component analysis
PDB	Protein data bank
PPI	Protein–protein interaction
pRCC	Papillary Renal Cell Carcinoma
RCC	Renal cell carcinoma
s-DIP	Secreted DIP
TCGA	The Cancer Genome Atlas
ZINC	ZINC Is Not Commercial
