# Supplementary material for: Differential Interactome Proposes Subtype-Specific Biomarkers and Potential Therapeutics in Renal Cell Carcinomas
Source: J Pers Med. 2021 Feb 23;11(2):158. doi: 10.3390/jpm11020158 (PMC7926666; doi:10.3390/jpm11020158)
Supplement: Supplementary file 1 [file jpm-11-00158-s001.pdf]

Table S1. List of Differentially Interacting Proteins (DIPs) in chRCC, ccRCC, pRCC

**DIPs specific to chRCC**	ABL1, ABTB2, ACADVL, ACBD3, ACE2, ACO2, ACTN1, ACTN3, ACTN4, ADRM1, AHNAK, AIFM1, AIP, AIRE, AK2, AKAP13, AKAP9, AKT1S1, AKT2, ALDH1B1, ALDH4A1, ALDOA, ALYREF, AMFR, ANXA2, ANXA5, AP1M1, APEX1, APH1A, APLF, APLP2, AQP1, ARAF, ARCN1, ARF1, ARHGDIA, ARIH1, ARRB1, ASAP1, ATF2, ATIC, ATP1B1, ATP1B3, ATP2B1, ATP6AP2, ATP6V1E1, ATP6V1G1, ATRX, ATXN1, AURKB, AXIN1, BAD, BAG6, BAP1, BECN1, BGN, BHMT, BLM, BMF,BMI1, BRAF, BRD8, BSG, BTF3, C1orf109, C1QBP, C6orf226, CALM2, CAPNS1, CARM1, CASP10, CAV1, CBR1, CBR3, CBX5, CBX7, CCNA2, CCNB1, CCNT1, CCS, CCT6A, CCZ1, CD151, CD74, CDC20, CDC25C, CDC37, CDC37L1, CDC6, CDH2, CDK11B, CDK18, CDK3, CDK5RAP2, CDK7, CDK9, CDR2, CEP162, CEP55, CHD1L, CHEK2, CHM, CHMP1B, CHMP2A, CHMP3, CHMP4A, CHMP4B, CHMP6, CHRM3, CIDEC, CITED2, CKAP5, CKS1B, CLASP1, CLINT1, CLOCK, CNNM3, CNTNAP1, COA3, COMMD1, COPA, COPB1, COPG1, COPS5, COX5A, COX6C, CPVL, CREB1, CREB3L1, CRTC2,CSDE1, CSNK2B, CSRNP2, CST3, CTNNA1, CTSB, CYB5B, CYC1, CYFIP2, CYLD, DACT1, DAD1, DAPK3,DCAF11, DCPS, DCTN2, DDX18, DERL1, DERL2, DIMT1, DLD, DLG1, DNAJB6, DNM1L, DNMT3A, DPYSL2, DSG2, DSTN, E2F1, EBNA1BP2, ECD, ECH1, ECHS1, ECI2, EDC3, EEF1B2, EEF1D, EEF2, EID1, EIF4A3, EMD, ENO3, EPC2, ERLIN1, ERLIN2, EZH2, FAF2, FAM53C, FANCG, FBXO7, FBXW7, FH, FKBP5, FLAD1, FLCN, FLNA, FLOT1, FOS, FOSB, FOSL2, FOXK1, FOXO3, FRMD6, FTH1, FUBP1, FUS, GALNT2, GAS2L1, GDI1, GET4, GLRX5, GNAI2, GOLGA5, GPI, GRAMD1C, GRB10, GSTA1, GTF2I, H3F3A, H3F3B, HADHA, HADHB, HARS, HDAC2, HDAC4, HDAC5, HDAC6, HERPUD1, HEXIM1, HGH1, HGS,HID1, HIPK1, HIRA, HIST3H2BB, HMGB1, HMGCR, HNRNPLL, HNRNPUL1, HSD17B10, HSPA1A, HSPA9,HSPE1, HTT, IER3, IFITM3, IGF1R, IGFBP7, INSIG2, IQCE, IRS2, ISG15, ITCH, ITGB5, IVNS1ABP, JUND, JUP, KANK1, KAT2A, KAT5, KCNJ16, KEAP1, KHDRBS1, KIAA0930, KIAA1217, KIF23, KIF5B, KLC1, KLC4, KRT18, KRT19, KRT8, LAMP1, LAMTOR2, LAP3, LAPTM4B, LEO1, LRBA, MACF1, MACROD1, MAF, MAGED2, MAP2K2, MAP3K1, MAP3K13, MAP3K2, MAP3K5, MAP3K7, MAPK6, MAPK7, MAPK8, MAPK9, MAPRE1, MAPT, MAZ, MCCC2, MCL1, MCM5, MDC1, MDH2, MDM4, MEF2A, MEF2D, MGMT, MGRN1, MIF, MLXIP, MORF4L2, MPRIP, MRM1, MRPL28, MRPS15, MRPS18B, MRPS34, MSH2, MSH6, MSN, MTA2, MUC1, NAP1L1, NASP, NAV1, NCOA3, NCOA6, NCOR2, NCSTN, NDUFA8, NDUFA9, NDUFAF1, NDUFB8, NDUFS5, NEDD4, NFE2L2, NFX1, NFYA, NME1, NME2, NOTCH1, NOTCH2NL, NPEPPS, NPLOC4, NPRL2, NPRL3, NR2C2, NR3C1, NRIP1, NUCB1, OAS3, OBSL1, OCIAD1, OS9, OTUD7B, PABPN1, PAFAH1B1, PAK4, PALB2, PARK7, PARP1, PCBP2, PCID2, PDCD4, PDCD6IP, PDPK1, PDZK1, PDZK1IP1, PEX5, PFN1, PGAM5, PGK1, PGM1, PGRMC1, PHB, PHB2, PHF12, PIAS1, PICALM, PIK3R1, PIK3R2, PIP5K1C, PKM, PLD2, PLS3, PML, PNN, POLE2, POLR2A, POMP, PPA1, PPFIBP1, PPIG, PPM1B, PPM1G, PPP1CB, PPP1R11, PPP1R13B, PPP1R37, PPP1R3C, PPP1R7, PPP1R8, PPP1R9B, PPP3CA, PPP4R2, PPP5C, PPT1, PQBP1, PRDX1, PRDX2, PRDX3, PRDX4, PRDX5, PRICKLE3, PRKACB, PRKAR2A, PRKDC, PRPF31, PSENEN, PSMA2, PSMA4, PSMA5, PSMA6, PSMA7, PSMB1, PSMB10, PSMB2, PSMB4, PSMB6, PSMB7, PSMB8, PSMB9, PSMC2, PSMC3, PSMC5, PSMC6, PSMD1, PSMD12, PSMD13, PSMD3, PSMD6, PSMD7, PSME1, PSME3, PTGES3, PTMA, PTOV1, PTPN14, PTPN23, PTPRK, PUF60, RAB11A, RAB11FIP2, RAB14, RAB1A, RAB1B, RAB5A, RAB5B, RAB5C, RAB7A, RABIF, RAD23A, RANBP3, RASSF5, RBBP7, RBFOX2, RBM39, RBM42,RBM7, RC3H1,RDX, REEP5, REEP6, RFC1, RIF1, RIOK1, RMDN3, RMND5A, RNF146, RNF187, RPA1, RPA2, RPL13A,RPL29, RPN2, RPS27A, RPS6KA1, RPS6KB1, RRAGA, RRAGC, RRAS,RRBP1, RRM2B, RUSC2, S100A10, SDC2, SEPT2, SEPT7, SEPT9, SET,

	SF3A2, SF3B1, SF3B2, SF3B3, SF3B5, SF3B6, SGCA, SGTA, SH2D4A, SIK2, SIRPA, SIRT1, SIRT7, SMAD2, SMAD3, SMARCA4, SMARCB1, SNRNP70, SNRPA, SNRPA1, SNX2, SOCS2, SOCS6, SOD2, SPAG5, SPARC, SQSTM1, SRPK1, SRRM1, SRSF3, SRSF7, STAC, STAMBP, STAT1, STAT6, STAU1, STIP1, STOM, STRAP, STUB1, STX11, STX3, STX4, STX8, SUPT5H, SYVN1, TBC1D1, TBC1D15, TBP, TCF4, TCF7L2, TDRD3, TENM1, TERF2, TERF2IP, TGOLN2, TMUB1, TNIP2, TONSL, TOP1, TOP2A, TOP2B, TP53BP1, TP53BP2, TPM3, TPM4, TRADD, TRAF6, TRAP1, TRAPPC2, TRIM14, TRIM21, TRIM23, TRIM27, TRIM8, TRIOBP, TRIP4, TRIP6, TSG101, TSPO, TTC4, TUBA1A, TUBB, TUFM, TXN, TXN2, TXNDC5, TYK2, U2AF2, UACA, UBA1, UBAC1, UBB, UBE2D1, UBE2D2, UBE2D3, UBE2D4, UBE2G1, UBE2G2, UBE2L6, UBE2M, UBE2W, UBE3A, UBE4A, UBXN2A, UCHL3, UCHL5, UCP3, UQCRC1, UQCRH, UQCRQ, URI1, USP14, USP15, USP17L2, USP30, USP43, USP50, VAMP8, VARS, VASP, VHL, VPS11, VPS35, WASL, WDR12, WWTR1, XRCC1, XRCC4, XRN1, YTHDC1, YWHAB, YWHAE, YWHAG, ZC3HAV1, ZDHHC17, ZFP91, ZNF622, ZNF830, ZNRF2, ZPR1
**DIPs specific to ccRCC**	ABCC2, AZIN1, B2M, BST2, CALU, CCDC106, CDT1, CENPA, CYB5R3, DDX3X, DKC1, DNAJB4, DTNBP1, ELF4, FBXW8, GABBR1, GIT2, GPS2, HLA-B, HSPBP1, IL32, IMMT, IRF1, LDOC1, MAPK3, MCM7, MCM9, MTF1, MTOR, NRP1, P4HA2, PDIA4, PEA15, PFDN2, PFKM, PHLPP1, PPIB, PRKCD, RGCC, RPS6KA3, RSL1D1, SCD, SDHA, TAF1, TAPBP, TNIP1, TOMM20, UBQLN1, USP2, ZNF668
**DIPs specific to pRCC**	CS, CUL3, DFFA, DHFR, EIF4A2, FLOT2, G6PD, GSTA2, GSTA4, HGF, IGBP1, ITGA3, LBH, LGALS8, MET, MME, MMGT1, MVP, PARP4, PGM2, PNPT1, PPM1A, RANBP9, SF3A3, SOCS1, TRAPPC1, TRAPPC12, TRAPPC2L, UNG
**Common DIPs for three subtypes**	AKT1, ATP1A1, BAG3, BAX, BCAP31, BCL2L1, BIN1, CCND1, CRYAB, CTNNB1, FBP1, FIS1, GRB2, HDAC1, HNRNPD, HNRNPH2, HSP90AB1, HSPA8, HSPB1, MYC, NCL, RPL21, RPL23, RPL27, RPL36, RPL37A, RPS4X, RPS9, RPSA, SNW1, VDAC1, VEGFA, YWHAQ
